# Evaluation of an Electronic Health Record System With a Disease Management Program and Health Care Treatment Costs for Danish Patients With Type 2 Diabetes

**DOI:** 10.1001/jamanetworkopen.2020.6603

**Published:** 2020-05-26

**Authors:** Ryan Pulleyblank, Giovanni Mellace, Kim Rose Olsen

**Affiliations:** 1 Danish Centre for Health Economics, Department of Public Health, University of Southern Denmark, Odense, Denmark; 2 Department of Business and Economics, University of Southern Denmark, Odense, Denmark

## Abstract

**Importance:**

Electronic health record (EHR) systems and disease management programs (DMP) are often promoted, but associated health care cost changes are not well understood.

**Objective:**

To evaluate the association between annual health care costs of patients with type 2 diabetes and the use of an electronic health record system with a disease management program (EHR/DMP) in general practice.

**Design, Setting, and Participants:**

This retrospective cohort study examined patients with type 2 diabetes in Denmark between January 1, 2008, and December 31, 2014, who attended practices that either used an EHR/DMP at a high level or never used the disease management system. An EHR/DMP system was rolled out across general practices in Denmark beginning in 2011 and was discontinued in 2014. Data were analyzed between March 2019 and March 2020.

**Main Outcomes and Measures:**

The main outcome was total health care costs, and the secondary outcomes were primary care, medication, nonhospital specialist, and hospital (total, outpatient, inpatient, and emergency) costs. Regression models were used to estimate EHR/DMP-associated percentage differences in patients’ annual health care treatment costs across health care treatment categories. All models included general practice–level fixed effects and patient-level controls. Two-part models examined robustness of estimated associations for hospital cost categories.

**Results:**

Of 33 970 patients included in the analysis, 15 953 (8016 [50.2%] male; mean [SD] age, 59.9 [13.3] years) attended 244 general practices that used the system at a high level, and 18 017 (9291 [51.6%] male; mean [SD] age, 60.0 [12.9] years) attended 344 general practices that had never used the system. Use of the EHR/DMP was associated with 3.2% higher (95% CI, 0.9%-5.6%) annual general practice treatment costs and with 6.4% lower (95% CI, –11.6% to –1.2%) annual hospital emergency visit costs. The associations between EHR/DMP use and annual total hospital (percentage difference, −0.8%; 95% CI, −7.5% to 5.7%) and total health care (percentage difference, −0.1%; 95% CI, −2.1% to 1.9%) cost changes were not statistically significant.

**Conclusions and Relevance:**

Among patients with type 2 diabetes, attendance at general practices that used an EHR/DMP was associated with a moderate increase in primary care costs and a reduction in emergency hospital visit costs but no significant change in total health care costs. Large health care cost savings associated with improved use of EHR-based disease management systems should not be expected to be realized in the short term.

## Key Points

**Question:**

Was use of an electronic health record (EHR) system with a disease management program associated with changes in health care costs of patients with type 2 diabetes?

**Findings:**

In this cohort study of 33 970 patients with types 2 diabetes, use of an EHR with a disease management program was associated with a small increase in patients’ treatment costs in general practice and a reduction in costs related to emergency hospital visits; however, there was no statistically significant change in cost overall.

**Meaning:**

These findings suggest that use of an EHR with a disease management program may be associated with improved health care process quality without increased overall treatment costs.

## Teaser

This cohort study assesses the association between use of an electronic health record (EHR) with a disease management system and health care costs for patients with type 2 diabetes in Denmark.

## Introduction

The implementation of electronic health record (EHR) systems has been a global shift in the delivery of health care over past decades. This has been partly associated with the implementation of the 2009 US Health Information Technology for Economic and Clinical Health (HITECH) Act,^1^ which initially offered financial incentives for the meaningful use of EHR systems and, after 2015, issued financial penalties for failure to do so.^1^ Particularly for patients with expensive chronic ambulatory care–sensitive conditions, such as diabetes, the use of EHR systems in primary care is regarded as having important potential to improve care provision through improved organization and exchange of patient information.^2^

The association of EHR systems in primary care with health care utilization has been investigated, with some evidence of improvements in health care delivery and outcomes. Evidence has shown that implementation of EHR systems may be associated with reductions in emergency visits and hospital admissions among patients with diabetes.^3^ Health care cost changes associated with EHR system use among different patient groups have not been well established.^3,4^

This study investigated the association of differences in health care costs of patients with type 2 diabetes (T2D) with the introduction and use of an EHR system with an integrated disease management program (DMP). Cost categories included total health care, primary general practice, medication, nonhospital specialists, and hospital (total, outpatient, inpatient, and emergency). To our knowledge, no patient-level estimates of health care cost differences associated with use of an EHR/DMP for patients with diabetes have been published. In comparison with measures such as general practice (GP) or hospital visits, focusing on cost outcomes has several potential advantages. For example, costs aggregate over the full treatment pathway are associated with disease severity^5^ and can be used to establish the cost-effectiveness of a program (in combination with information on the administrative costs of preparing and maintaining the system).

## Methods

### Study Design

This retrospective cohort study of the association between EHR/DMP use and health care costs considered patients with T2D between January 1, 2008, and December 31, 2014. Approval for conducting the study was provided by the Danish Data Protection Agency. Informed consent was waived because all data were from anonymized administrative registries (Danish Act on Research Ethics Review of Health Research Projects §14, section 2). Analyses were conducted between March 2019 and March 2020. This study followed the Strengthening the Reporting of Observational Studies in Epidemiology (STROBE) reporting guideline.

### Setting

Denmark is a northern European country with a population of 5.8 million.^6^ Denmark provides an extensive publicly financed health care system, and Danish administrative registers provide significant opportunities for health care services research.^7^ Proportional to gross domestic product, health care spending in Denmark (10.4%) is above average among nations in the Organization for Economic Cooperation and Development (9.0%) but below the US (17.2%).^8^ Out-of-pocket expenditure accounts for 14% of national health care expenditure, voluntary insurance covers 2%, and the remaining 84% is publicly financed.^8^ The overall prevalence of diabetes in Denmark is approximately 4.4%.^9^

Primary care in Denmark is mostly delivered by self-employed general practitioners, who have gatekeeper responsibilities, controlling access to (almost all) non–hospital-based specialist and hospital-based care.^10^ The national GP contract is renegotiated approximately every 3 years, and compensation is through a combination of capitation and fee-for-service. As of 2020, there were 69 hospitals in Denmark,^11^ and almost all were publicly owned (97% of beds), with doctors paid by salary.^12^ Hospitals are remunerated based on a mixture of diagnosis-related group payments and block grants.^13^ Non–hospital-based specialists are paid based on fee-for-service tariffs. Prescription medications are covered through public health insurance and operationalized with a stepped system of decreasing copayments (covered at 100% beyond a patient’s annual contribution equal to $632 in 2018).^14,15^

### EHR/DMP

The national contract agreed in December 2010 required GPs to enroll in the GP-facing EHR/DMP within a 3-year period (2011-2013).^16,17^ In brief, the system required GPs to code patient visits (using the International Classification of Primary Care, version 2 standard) and also took other clinical information as inputs (eg, laboratory results, prescriptions). All patient visits to the GP were meant to be coded in the registry. The system included disease management modules for specific groups of patients (eg, diabetes, coronary heart disease, chronic obstructive pulmonary disease, and anxiety and depression). The diabetes module included tracking of health measures, including glycated hemoglobin (HbA_1c_) levels, blood pressure, body mass index, cholesterol level, and albuminuria, with color-coded indicators to highlight where patients were missing targets. In addition, the system enabled GPs to observe their performance compared with peers across local, regional, and national levels. Examples of comparison metrics in the diabetes module included rates of patients receiving annual control visits, patients with HbA_1c_ level greater than or equal to 53 mmol/mol (≥7.0%) who were receiving antidiabetic medications, and patients with systolic blood pressure greater than or equal to 140 mm Hg who were receiving antihypertensive medications. The system was developed and managed by the Danish General Practice Quality Unit, and GPs did not pay to use the system. The system has been previously described in detail.^16,18^

Although enrollment was mandatory, there was some exogeneity of implementation owing to technical compatibility variation among GPs’ information technology systems. Exact timing of enrollment and the extent to which the system was used was determined by the GP. Owing to the extent of data that were being collected, which was determined to exceed what Danish data privacy laws allowed at the time, the system was terminated in September 2014.^19^ Although clinical data were deleted, metadata identifying the extent to which GPs used the system were retained.

### Study Population

The study population consisted of adults (aged ≥18 years) with T2D who attended GP practices that either never used the EHR/DMP for care of patients with diabetes (control) or used the system at a high level beginning in 2012 (treatment). Previous research identified the GPs that achieved high-level use of the system in 2011 as highly selected (ie, early adopters) that were believed to have a particular interest in diabetes treatment, and participation in the EHR/DMP in 2012 was believed to be random and largely determined by the information technology system used.^17^ Restricting analysis of the treated group to those attending GPs meeting criteria in 2012 minimized selection bias. Patients were identified algorithmically based on records of hospitalization with diabetes diagnoses or prescriptions for diabetes-specific medications. Responsibility for care of patients with type 1 diabetes in Denmark resides with specialist outpatient clinics, and thus these patients were excluded. The study period (2008-2014) covered a period before the system’s introduction until when the system was closed. To avoid composition bias, the analysis was restricted to patients identified as diabetic by 2008 who remained patients at the same GP throughout the study period and did not die.

### Data

An anonymized identifier was used to cross-link individual level data from relevant Danish administrative registers. These include health care registries (the National Patient Register,^20^ the National Health Service Register,^21^ and the Prescription Drug Register^22^) and registers with individual socioeconomic variables from Statistics Denmark.^23,24,25^ Data on program participation were obtained from the Danish General Practice Quality Unit.

### Cost Outcomes

All health expenditures covered through public funding are captured in Danish administrative registries and can be cross-linked at the individual patient level. The main outcome was percentage differences in patients’ total annual health care costs associated with EHR/DMP use at their GP. Secondary outcomes included associated primary care treatment, medication, nonhospital specialist, and hospital (total, outpatient, inpatient, and emergency) costs. All costs were originally measured in Danish krone and inflated to a common base year (2016) using inflation factors provided by the Danish Association of Regions, a government body responsible for coordinating health care provision and policy. All costs have been converted to US dollars using the historical Danish krone to US dollar exchange rate on July 1, 2016.^26^

### Treatment Exposure

Untreated patients were those attending control GPs, defined as GPs that never accessed the diabetes disease management module and never exceeded a coding rate of 10% of patient visits. Treated patients were those attending GPs known to access the diabetes disease management module and sustainably achieved a median annual rate of coding 70% of patient visits starting in 2012. Thus, the exposure variable indicated treated patients from 2012 through 2014. The 70% coding rate was chosen because GPs achieving this rate were given a special designation as a sentinel practice, interpreted as being compliant with the program.^27^

### Control Variables

By definition, fixed-effects models control for time-invariant unobserved confounder variables, including those affecting selection into the EHR/DMP treatment group. Including GP-level fixed effects reduced bias that may have been caused by GP-level variation between treatment and control GPs.

A rich set of patient-level control variables capturing a range of health and sociodemographic characteristics believed to be potentially associated with variation in health care demand, access, and treatment costs were included. These characteristics included age, sex, years of diabetes, and Charlson comorbidity index score as well as sociodemographic measures, including educational level, income, employment status, cohabitation status, number of adult children, and immigration status. These variables capture aspects of patients’ health and social status as well as available social support.

### Statistical Analysis

We investigated the association between annual health care costs of patients with T2D and their GP’s use of the EHR/DMP using regression models including GP-level fixed effects. Two sets of models were estimated. The first set of models used log-transformed annual costs for all health care costs. Second, for hospital cost categories that had high proportions of 0 annual costs, 2-part models were estimated as robustness checks. The first part was a logistic regression of probability of hospital costs, and the second part was a conditional regression on positive log-transformed hospital costs. All models controlled for secular annual trends and included a variable for exposed patients during the exposure period (2012-2014), which captures the association of the EHR/DMP. With log-transformed costs, the estimated parameter of association between EHR/DMP use and cost was interpreted as a percentage difference. Model specifications can be found in the eMethods in the Supplement.

Sensitivity analyses were conducted in which 60% and 80% patient visit coding rate thresholds were used for defining treatment group GPs. The 80% treatment group was a subset of the 70% treatment group, and the 70% treatment group of the main analysis was a subset of the 60% treatment group. The control group remained the same in all analyses. All hypotheses tests were 2-sided using a significance threshold of *P* ≤ .05. Analyses were conducted using Stata, version 15 (StataCorp).

## Results

### Study Population

Of 33 970 patients included in the analysis, 15 953 (8016 [50.2%] male; mean [SD] age, 59.9 [13.3] years) attended 244 general practices that used the system at a high level, and 18 017 (9291 [51.6%] male; mean [SD] age, 60.0 [12.9] years) attended 344 general practices that had never used the system. The data set only includes patients which interact with the Danish health care system within a given year; thus, not all patients were observed in each year.

Baseline characteristics are presented in Table 1. Similar distributions were observed for age at baseline, sex, educational level, cohabitation status, number of adult children, income, occupational status, and immigration status. Furthermore, health status variable of mean time living with diabetes (6.3 [4.0] years vs 6.2 [3.9] years) and Charlson comorbidity index scores (1.3 [1.4] vs 1.3 [1.4]) were similar between the control and treatment groups.

**Table 1.** Baseline Characteristics by Patient Group^a^

Characteristic	Control group (n = 18 017)	Treatment group (n = 15 953)
Age, mean (SD), y	60.0 (12.9)	59.9 (13.2)
Male, No. (%)	9291 (51.6)	8016 (50.2)
Duration of diabetes, mean (SD), y	6.3 (4.0)	6.2 (3.9)
Charlson comorbidity index score, mean (SD)	1.3 (1.4)	1.3 (1.4)
Income, mean (SD), 2016 US dollars	32 282 (34 679)	32 505 (37 859)
Highest educational level, No. (%)		
Secondary	7479 (41.5)	6617 (41.5)
Undergraduate	1709 (9.5)	1627 (10.2)
Postgraduate	577 (3.2)	477 (2.9)
Occupation, No. (%)		
Employed	6362 (35.3)	5916 (37.1)
Student	39 (0.2)	47 (0.3)
Retired	10 525 (58.4)	9128 (57.2)
Lives alone, No. (%)	5384 (29.9)	4443 (27.9)
Adult children, mean (SD), No.	1.6 (1.4)	1.6 (1.3)
Immigrant, No. (%)		
First generation	2011 (11.2)	1586 (9.9)
Second generation	64 (0.4)	31 (0.2)
GP practice list size, mean (SD)	2499 (1681)	2876 (1449)

Abbreviation: GP, general practice.

^a^ Patients treated at GPs using an electronic health record disease management program were the treatment group and patients treated at GPs that never used an electronic health record disease management program were the control group.

Mean baseline costs per patient by annual health care cost category are presented in Table 2. Annual patient-level data covering several categories of health care costs were examined in this analysis, including general practice, medication, non–hospital-based specialist care, hospital (outpatient, inpatient, emergency, and total), and total health care costs. Baseline mean annual costs of health care treatment at patients’ GPs accounted for 7% of total annual health care costs. Hospital costs accounted for 64%, including 21% classified as emergency.

**Table 2.** Baseline Annual Health Care Costs Per Patient by Patient Group^a^

Cost category	Cost, mean (SD), 2016 US dollars	
	Control group (n = 18 017)	Treatment group (n = 15 953)
Total health care	4728 (9024)	4686 (8762)
Primary care, general practice	324 (242)	342 (246)
Medication	998 (1521)	966 (1356)
Nonhospital specialists	327 (496)	332 (541)
Total hospital	3059 (8609)	2975 (8393)
Hospital outpatient	1178 (3528)	1158 (2920)
Hospital inpatient	1882 (7112)	1816 (7156)
Hospital emergency^b^	1003 (4674)	950 (3896)

^a^ Patients treated at general practices using an electronic health record disease management program were the treatment group and patients treated at general practices that never used an electronic health record disease management program were the control group.

^b^ Emergency costs included both inpatient and outpatient costs.

Unadjusted mean annual health care costs between 2008 and 2014 for the treated and control patients are presented in the Figure. As expected of an aging patient cohort, there was an overall increasing trend throughout the observation period. Total annual health care expenditures increased by 42%. Annual total hospital expenditures increased by 60%, of which emergency hospital expenditures increased by 75%. Mean annual medication costs were up 11%.

**Figure.**
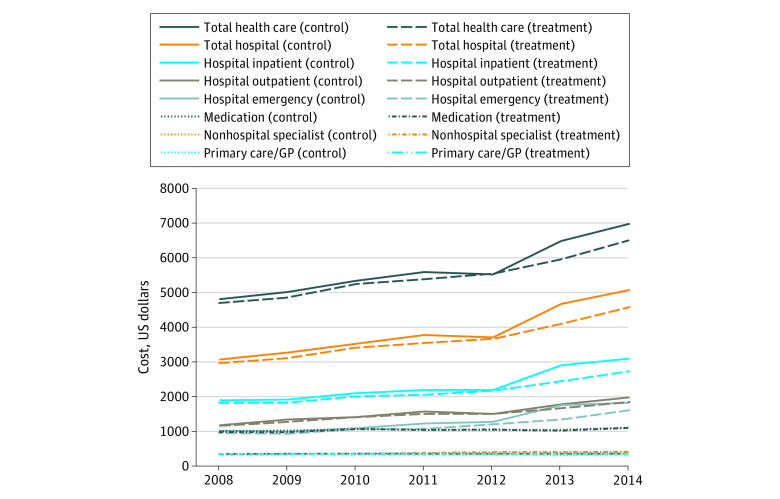
Unadjusted Mean Annual Health Care Costs Between 2008 and 2014 for the Patients Treated at General Practices (GPs) Using an Electronic Health Record Disease Management Program and Control Patients Patients treated at GPs using an electronic health record disease management program were the treatment group and patients treated at GPs that never used an electronic health record disease management program were the control group.

### Main Results

Estimated mean annual health care cost percentage differences associated with the use of the EHR/DMP are presented in Table 3. There was no association between EHR/DMP use and changes in annual total hospital (−0.8%; 95% CI, −7.5% to 5.7%) and total health care costs (−0.1%; 95% CI, −2.1% to 1.9%). There was a positive association between EHR/DMP use and annual treatment costs in general practice (3.2%, 95% CI, 0.9% to 5.6%) and a negative association with annual emergency hospital visit costs (–6.4%, 95% CI, –11.6% to –1.2%). For hospital cost categories, which had high rates of 0 annual cost observations, robustness checks based on 2-part models were similar.

**Table 3.** Association Between EHR/DMP Use and Annual Health Care Cost Differences^a^

Cost category	Fixed-effects models		2-Part models^b^	
	Estimate (SE)	*P* value	Estimate (SE)	*P* value
Total health care	–0.001 (0.010)	.99	NA	NA
Primary care, general practice	0.032 (0.012)	.008	NA	NA
Medication	–0.006 (0.011)	.56	NA	NA
Nonhospital specialists	0.031 (0.020)	.12	NA	NA
Total hospital	–0.008 (0.034)	.82	–0.005 (0.034)	.88
Hospital outpatient	0.005 (0.033)	.88	0.007 (0.033)	.84
Hospital inpatient	–0.026 (0.030)	.37	–0.024 (0.029)	.41
Hospital emergency^c^	–0.064 (0.027)	.02	–0.057 (0.027)	.03

Abbreviations: EHR, electronic health record; DMP, disease management program; NA, not applicable.

^a^ A 70% threshold was used.

^b^ Average marginal effects are reported.

^c^ Emergency costs included both inpatient and outpatient costs.

Results based on 60% and 80% coding thresholds for identifying the treatment groups are reported in eTables 1 and 2 in the Supplement). The positive association between EHR/DMP use and annual primary care costs was significant when considering the fixed effects models for both the 60% (3.8%; 95% CI, 1.4%-6.2%) and 80% (3.1%; 95% CI, 0.4%-5.8%) treatment groups. The negative association between EHR/DMP use and annual hospital emergency costs was significant when considering the fixed effects models for both the 60% (–6.5%; 95% CI, –11.6% to –1.4%) and 80% (–5.8%; 95% CI, –11.5% to –0.1%) treatment groups. The 2-part model at the 80% treatment threshold was not associated with emergency hospital visit costs.

## Discussion

In this study, use of the EHR/DMP was positively associated with mean annual general practice costs and negatively associated with mean annual emergency hospital costs, but there were no associations with difference in total hospital or total health care costs.

Although a positive association was observed, to put the estimated 3.2% ($9.13) positive association into perspective, the basic fee paid to a Danish GP for an office consultation was $20.46 and the fee paid to GPs for conducting an HbA_1c_ blood test was $17.17 in 2016.^28^ Although these findings suggest that GPs that used the EHR/DMP may have changed some treatment behaviors, they also suggest that the typical patient at a GP clinic using the EHR/DMP may not have received substantially different treatment than they would have been without the system. Given that the system was designed to identify patients for whom extra attention was appropriate, if care was generally quite good, patients would not have a marked increase in primary care expenditure on average. A likely explanation is that most patients did not receive any increase in attention, but a small percentage who had been undertreated received additional appropriate attention. Use of this EHR/DMP has previously been shown to be associated with a reduction in inequality of access in general practice among patients with diabetes.^17^

The finding of a negative association between EHR/DMP use and mean annual emergency hospital costs but no association with differences in total hospital costs suggests that hospital visits were more likely to have been planned rather than emergency. That is, using the EHR/DMP may have been associated with GPs more effectively referring patients with diabetes to the hospital as appropriate. This can be interpreted as an improvement in process quality for patients attending these GPs. If the procedural quality of the health care system increased without increasing overall health care costs, use of the EHR/DMP my be considered to be associated with increased efficiency of the Danish health care system for affected patients with diabetes. However, this evidence does not indicate what may have happened with other groups of patients.

### Limitations

This study has limitations. Diabetes is recognized as an ambulatory care–sensitive condition, meaning that the quality of primary care for patients with diabetes is recognized as an important factor associated with rates of hospitalizations.^29^ Emergency visits and inpatient hospitalizations are expensive but relatively infrequent, and the full ability of an EHR/DMP based in primary care to affect overall quality of care would be expected to emerge over a long period. To identify statistically significant changes in health care cost categories that are further removed from the GP setting where the system existed, evidence accumulated over many years would be required. The EHR/DMP was discontinued in late 2014, and therefore we only observed 3 years of EHR/DMP use. Stronger evidence of the system’s consequences may have emerged had the system not been discontinued suddenly. Potential net cost savings from improved use of EHR-based disease management systems should not be expected in the short term.

Although extensive clinical data were collected in the EHR system, clinical outcomes associated with the use of the system could not be investigated. The clinical data were deleted when the system was discontinued.

The effects of any organizational intervention is relative to the baseline organizational structure. At the beginning of the period when the Danish GPs became obliged to implement the EHR, a variety of computer systems were used for administration, some of which were incompatible with the implementation of the EHR. Furthermore, the fundamental relationships between particular general practices and computerized medicine vary between practices, affecting the potential effects of EHR/DMP use. By using fixed-effects models that controlled for unobserved time-invariant factors and by excluding highly motivated and selected early-adopter GPs, selection bias was substantially reduced in the analyses. However, because the treatment groups were not randomized, the results should not be interpreted causally. The generalizability of findings of health care cost changes associated with use of an EHR/DMP system is likely to be greater in countries with strong centrally organized, publicly financed health care systems.

## Conclusions

Use of an EHR system with a built-in diabetes disease management module was associated with slightly higher mean annual treatment costs in GP and lower mean annual costs for emergency hospital visits for patients with T2D. However, given the absence of significant reductions in total hospital costs, the reduction in emergency hospital costs could only be interpreted as indicating a procedural improvement in quality of care. No significant overall health care treatment cost changes associated with EHR/DMP use were observed.

## Notes

Supplement. **eMethods.** Model Specifications **eTable 1.** Association between EHR/DMP Use and Annual Health Care Cost Differences (%/100) (60% Threshold) **eTable 2.** Association between EHR/DMP Use and Annual Health Care Cost Differences (%/100) (80% Threshold)
