# Supplementary material for: Evaluation of an Electronic Health Record System With a Disease Management Program and Health Care Treatment Costs for Danish Patients With Type 2 Diabetes
Source: JAMA Netw Open. 2020 May 26;3(5):e206603. doi: 10.1001/jamanetworkopen.2020.6603 (PMC7251448; doi:10.1001/jamanetworkopen.2020.6603)
Supplement: Supplement. — eMethods. Model Specifications eTable 1. Association between EHR/DMP Use and Annual Health Care Cost Differences (%/100) (60% Threshold) eTable 2. Association between EHR/DMP Use and Annual Health Care Cost Differences (%/100) (80% Threshold) [file jamanetwopen-3-e206603-s001.pdf]

Supplementary Online Content

Pulleyblank R, Mellace G, Olsen KR. Evaluation of an electronic health record system with a disease management program and health care treatment costs for Danish patients with type 2 diabetes. *JAMA Netw Open*. 2020;3(5):e206603. doi:10.1001/jamanetworkopen.2020.6603

eMethods. Model Specifications
eTable 1. Association between EHR/DMP Use and Annual Health Care Cost Differences (%/100) (60% Threshold)
eTable 2. Association between EHR/DMP Use and Annual Health Care Cost Differences (%/100) (80% Threshold)

This supplementary material has been provided by the authors to give readers additional information about their work.

© 2020 Pulleyblank R et al. *JAMA Network Open.*

eMethods. Model Specifications

Fixed-Effects Models

Where *X* represents a set of (*K*) controls, given patient *i* attending GP *j*, in year *t*, the models are defined as:

$$\ln HealthCareCost_{ijt} = \mu + \sum_{k=1}^{K} \pi_k X_{it}^{k} + \beta DMP_i + year_t + GP_j + \epsilon_{ijt}$$

where,

$$Period = \begin{cases} 0 \text{ if year} < 2012 \\ 1 \text{ if year} \geq 2012 \end{cases}$$

$$GPType_i = \begin{cases} 0 \text{ if patient } i \text{ attended General Practice where DMP was not ever used} \\ 1 \text{ if patient } i \text{ attended General Practice where DMP was used} \end{cases}$$

$$DMP_i = GPType_i * Period$$

and control variables X^k are coded as:

Age: dummy coded: 18-29 (reference) / 30-39 / 40-49 / 50-59 / 60-69 / 70-79 / 80-89 / 90+
Diabetes age: coded as linear count
Gender: dummy coded: female (reference) / male
Highest Education: dummy coded: primary (reference) / secondary / undergraduate university / post-graduate university
Occupation status: dummy coded: unemployed (reference) / employed / student / retired
Income: coded as linear count
Cohabitation status: dummy coded: cohabits (reference) / lives alone
Adult children: coded as linear count
Immigration status: dummy coded: Danish (reference) / first-generation / second-generation
Charlson Comorbidity Index: dummy coded: 0 (reference) / 1-3 / 4-6 / 7-9 / 10+

Dummy coding (e.g. of age and Charlson Comorbidity Index) avoids any assumptions of smooth linear relationships between outcome (i.e. log-transformed cost) and control variable value.

The estimated EHR/DMP associated impact on healthcare costs is captured in the β parameter estimate.

Standard errors were clustered by GP.

© 2020 Pulleyblank R et al. *JAMA Network Open.*

Two-Part Models for Hospital Cost Outcomes

With the same control variables and GP-fixed effects as for the standard Fixed-Effects models.

Part-one is a logistic regression on positive (log-transformed) cost outcomes.

Part-two is a linear regression *conditional* upon having positive costs.

The estimated EHR/DMP associated impact on healthcare costs is estimated as the average marginal effect associated with the DMP_i indicator.

Standard errors were clustered by GP.

© 2020 Pulleyblank R et al. *JAMA Network Open.*

eTable 1. Association between EHR/DMP Use and Annual Health Care Cost Differences (%/100) (60% Threshold)

	Fixed-Effects Models			2-Part Models[a]		
Cost Category	Estimate	(SE)	*P* value	Estimate	(SE)	*P* value
Total Healthcare	0.000	(0.009)	0.98			
Primary Care - GP	0.038**	(0.012)	0.001			
Medication	-0.005	(0.011)	0.67			
Nonhospital Specialists	0.038	(0.020)	0.05			
Total Hospital	-0.019	(0.032)	0.55	-0.017	(0.032)	0.59
Hospital Outpatient	-0.008	(0.031)	0.79	-0.007	(0.031)	0.81
Hospital Inpatient	-0.035	(0.029)	0.22	-0.033	(0.027)	0.24
Hospital Emergency[b]	-0.065*	(0.026)	0.01	-0.058*	(0.026)	0.02

[a]Average marginal effects reported. [b]Emergency costs include both inpatient and outpatient costs. * p ≤ 0.05, ** p ≤ 0.01.

eTable 2. Association between EHR/DMP Use and Annual Health Care Cost Differences (%/100) (80% Threshold)

	Fixed-Effects Models			2-Part Models[a]		
Cost Category	Estimate	(SE)	*P* value	Estimate	(SE)	*P* value
Total Healthcare	0.005	(0.011)	0.65			
Primary Care - GP	0.031*	(0.014)	0.02			
Medication	-0.005	(0.012)	0.66			
Nonhospital Specialists	0.036	(0.023)	0.11			
Total Hospital	-0.019	(0.037)	0.60	-0.020	(0.037)	0.60
Hospital Outpatient	-0.013	(0.036)	0.73	-0.013	(0.036)	0.72
Hospital Inpatient	-0.011	(0.033)	0.74	-0.010	(0.032)	0.76
Hospital Emergency[b]	-0.058*	(0.029)	0.04	-0.052	(0.029)	0.07

[a]Average marginal effects reported. [b]Emergency costs include both inpatient and outpatient costs. * p ≤ 0.05, ** p ≤ 0.01.

© 2020 Pulleyblank R et al. *JAMA Network Open.*
